# Supplementary material for: Generative modelling of the thalamo-cortical circuit mechanisms underlying the neurophysiological effects of ketamine
Source: Neuroimage. 2020 Nov 1;221:117189. doi: 10.1016/j.neuroimage.2020.117189 (PMC7762824; doi:10.1016/j.neuroimage.2020.117189)
Supplement: Supplementary file 1 [file mmc1.docx]

**Supplementary Materials: Thalamo-cortical oscillatory model for ketamine – Shaw et al 2019**

Observation model.

The purpose of the observation model is to transform the integrated channel-by-time voltage series into a local field potential (LFP)-like spectrum, for comparison to the MEG virtual sensor spectrum. In this study we assume that the MEG virtual sensor data is a weighted sum of the voltage series of the principal (pyramidal) cells. Due to the complex oscillatory nature of the membrane series, where the timeseries of each population demonstrates oscillations of multiple frequencies, we employed Dynamic Mode Decomposition (DMD) to separate the contributing population timeseries into a set of frequency-specific modes (DMD is introduced in (Schmid, 2010) and has been used for neural data previously, for example in (Brunton et al., 2016)). The single-channel output spectrum is computed by parameterised (n=4) weighted sum of the smoothed Fourier transformed mode data. Finally, an electrode gain parameter, L, was applied. Supplementary figure 1 summarises the integration and observation steps and supplementary figure 2 illustrates the DMD routine.


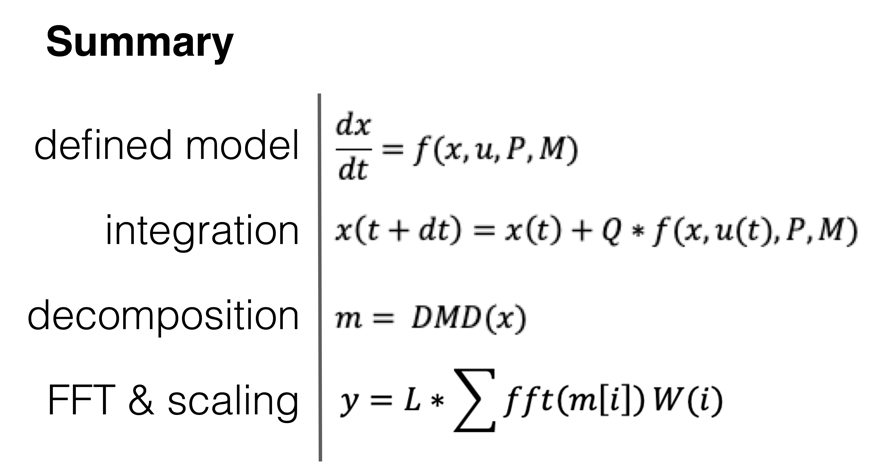


Supplementary Figure S1. Summary of the generative model specification, integration, decomposition and spectral output steps.


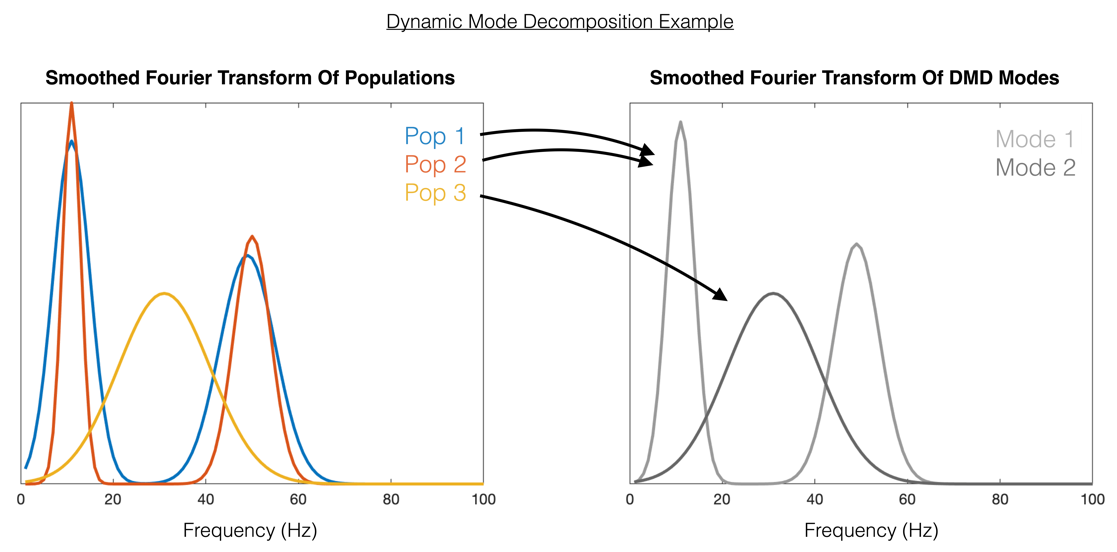


Supplementary Figure 2. Depicts the use of dynamic model decomposition (DMD) to compute a set of modes (‘Mode 1/2') from the complex oscillatory signals (‘Pop 1 to 3’) of the contributing populations (in Fourier space for clarity).

| Channel | ion | Reversal (mV) | Decay rate (ms) |
| --- | --- | --- | --- |
| Potassium Leak | K | -70 | ~ |
| AMPA | Na | 60 | 4 |
| NMDA | Ca | 10 | 100 |
| GABAA | Cl | -90 | 16 |
| GABAB | ~ | -100 | 200 |
| M | K | -70 | 160 |
| H | Non  selective | -30 | 100 |

Supplementary Table S1. Reversal potentials and receptor decay constants for each channel in the model.


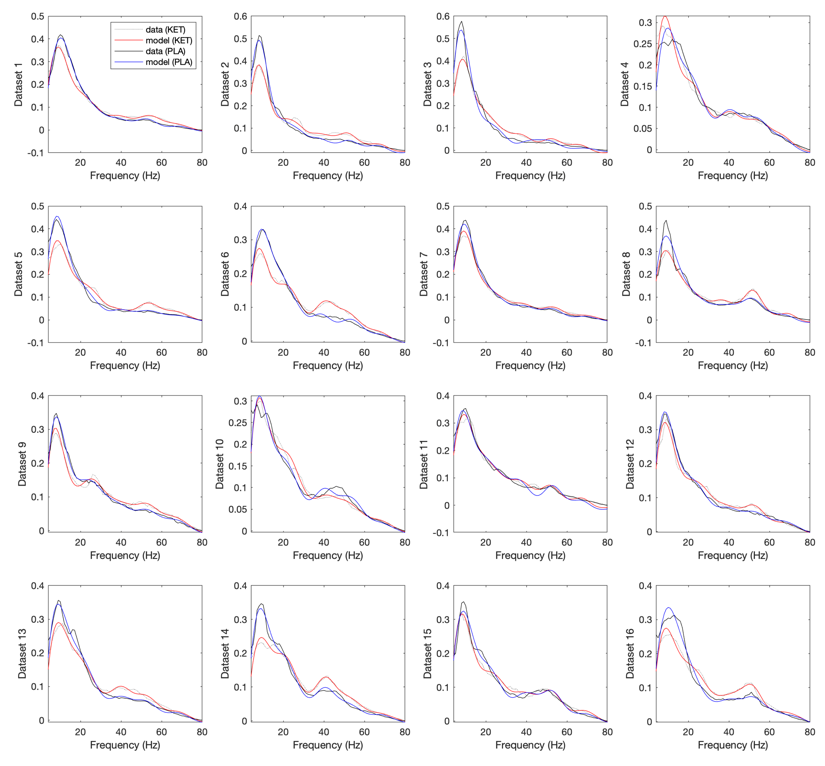


Supplementary Figure S3. Individual dataset model fits.

| **Parameter** | **Description** | **Prior Mean** | **Variance** |
| --- | --- | --- | --- |
| H / Hn (41 parameters) (see Sup. Fig. 4) | Intrinsic connection strengths | Excitatory = 4  Inhibitory = 8 | 1/8 |
| TC | Log scale parameter on Receptor Rates in supp table 1 | 0 | 1/8 |
| Input (u) | D.C to Relay population | 2 | 1/8 |
| Delays (D) | Thal🡪Cort Cort🡪Thal | 3 ms [0] 8ms [0] | 1/8 |
| Weights [J] | Weightings on frequency-modes (x4) | 1 | 1/8 |
| Electrode Gain [L] | Scales output spectrum | 1 | 1/8 |

Supplementary Table S2. Parameters with non-zero variances – i.e. which varied during model fitting.


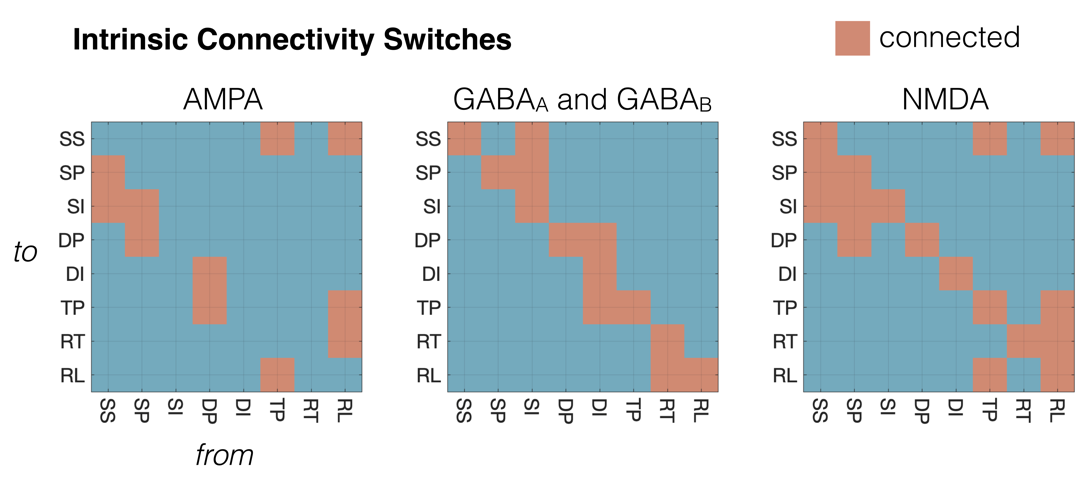


Supplementary Figure S4. Intrinsic Connectivity Switches for AMPA, NMDA and GABA-A/B. Population abbreviations: **SS** = L4 Spiny Stellates, **SP** = L2/3 (Superficial Layer) Pyramidal Cells, **SI** = (Superficial Layer) Inhibitory Interneurons, **DP** = L5 (Deep Layer) Pyramidal Cells, **DI** = L5 (Deep Layer) Inhibitory Interneurons, **TP** = L6 (Thalamic Projection) Pyramidal Cells, **RT** = Thalamic Reticular Cells, **RL** = Thalamic Relay Cells.


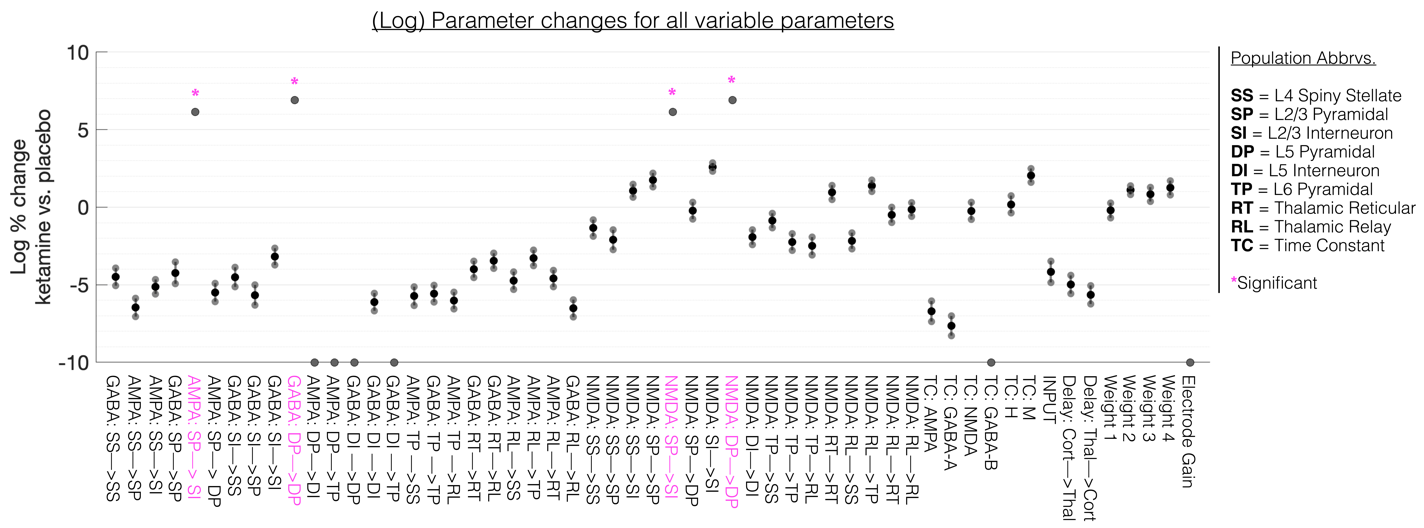


Supplementary Figure S5. Summary of the mean drug-induced changes in parameter estimates for all variable parameters. Those in pink are significant and survive multiple comparison correction, and correspond to those in figure 5 of the main text.
